# Impact of the COVID-19 Pandemic on Routine Childhood Vaccination Coverage in Ecuador From 2019 to 2021: Comparative Analysis

**DOI:** 10.2196/75293

**Published:** 2025-10-17

**Authors:** Jose Sanchez, Alejandro Arjuna Rodriguez Sr, Kimberlly Pamela Montenegro Cuello Sr

**Affiliations:** 1 Faculty of Health Sciences and Human Well-being, Universidad Indoamérica, Avenida Machala y Sabanilla, La PraderaQuito, 170509, Ecuador, 593 0984663224; 2 Faculty of Health Sciences "Eugenio Espejo", Universidad UTE, Quito, Ecuador

**Keywords:** COVID-19 pandemic, vaccination coverage, Ecuador, immunization, routine vaccination, health disparities, vaccine hesitancy

## Abstract

**Background:**

The COVID-19 pandemic disrupted essential health care services globally, including routine childhood immunization programs. Ecuador faced significant challenges in maintaining vaccination coverage during this period.

**Objective:**

The aim of this study is to analyze the impact of the COVID-19 pandemic on routine childhood vaccination coverage in Ecuador by comparing prepandemic (2019) and pandemic (2020‐2021) data.

**Methods:**

This retrospective observational study analyzed vaccination coverage data from the Ministry of Public Health of Ecuador and demographic data from the National Institute of Statistics and Censuses. We examined routine childhood vaccination coverage for children under 24 months across all 24 provinces. Statistical analyses were performed using SPSS (version 28.0), including descriptive statistics and comparative analysis. Coverage rates were calculated as percentages of children in target age groups receiving recommended doses.

**Results:**

A significant decline in routine childhood vaccination coverage was observed during the pandemic. BCG vaccine coverage decreased from 86.4% in 2019 (n=286,569) to 80.7% in 2020 (n=266,961) and 75.3% in 2021 (n=248,812). Pentavalent vaccine third dose coverage dropped from 85.0% to 68.0% across the same period. The most dramatic decline was seen in measles-mumps-rubella vaccine second dose coverage, falling from 75.7% in 2019 to 58.4% in 2021. Coastal and highland provinces experienced the most severe reductions, with approximately 137,000 fewer doses administered in 2020 compared to stable prepandemic levels.

**Conclusions:**

The COVID-19 pandemic significantly impacted routine childhood vaccination coverage in Ecuador, with sustained declines through 2021. Regional disparities were evident, with vulnerable populations facing greater challenges accessing immunization services. Urgent interventions, including catch-up campaigns and strengthened health systems, are needed to restore coverage levels and prevent outbreaks of vaccine-preventable diseases.

## Introduction

### Background and Global Context

The COVID-19 pandemic emerged as an unprecedented global health crisis, fundamentally disrupting health care systems and essential services worldwide [1]. Beyond the direct morbidity and mortality caused by SARS-CoV-2, the pandemic created far-reaching consequences for routine health care delivery, particularly impacting childhood immunization programs that are critical for preventing infectious diseases and maintaining population health [2,3].

Global evidence demonstrates substantial disruptions to vaccination services during the pandemic. The World Health Organization reported that at least 68% of countries experienced disruptions to childhood immunization programs, with low- and middle-income countries disproportionately affected [4]. These disruptions resulted from multiple factors including health care worker redeployment, supply chain interruptions, physical distancing measures, and reduced health care–seeking behavior due to fear of COVID-19 transmission [5,6].

### Impact on Low- and Middle-Income Countries

In low- and middle-income countries, where health care infrastructure may be fragile and resources limited, the pandemic exacerbated preexisting challenges in vaccination delivery [7]. Countries in Latin America and the Caribbean faced particular difficulties, with studies showing that COVID-19 containment measures led to significant reductions in routine immunization coverage across the region [8]. Castro-Aguirre et al [9] conducted a comprehensive analysis of 39 countries and territories in Latin America and the Caribbean, finding significant reductions in diphtheria-pertussis-tetanus (DTP) vaccine coverage in 79% of assessed regions.

### Ecuador’s Prepandemic Vaccination Context

Ecuador, a South American country with diverse geographical regions and varying levels of health care access, operated a national immunization program that faced coverage challenges even before the pandemic [10]. The country’s immunization system demonstrated disparities across different geographical regions and socioeconomic groups, with rural and Indigenous populations often experiencing lower vaccination rates [11].

Prior to 2020, Ecuador’s routine childhood vaccination program included vaccines against tuberculosis (BCG), diphtheria-pertussis-tetanus-hepatitis B-*Haemophilus influenzae* type b (pentavalent), pneumococcal disease, poliovirus, rotavirus, measles-mumps-rubella, yellow fever, and varicella [12]. Coverage rates varied significantly across provinces, reflecting the country’s geographical challenges and socioeconomic disparities [13].

### Pandemic Impact in Ecuador

As of July 2021, only 57% of Ecuador’s population had received the first COVID-19 vaccine dose, highlighting significant challenges in reaching underserved populations in remote areas [14]. The pandemic’s impact on routine childhood vaccination was particularly concerning, given the potential for vaccine-preventable disease outbreaks in an already vulnerable population [15].

The implementation of movement restrictions, health care system overwhelm, and resource reallocation to COVID-19 response efforts created substantial barriers to routine immunization services [16]. Health care facilities experienced reduced capacity, parents delayed or avoided medical visits due to infection fears, and supply chains faced significant disruptions [17].

### Study Rationale and Objectives

Understanding the specific impact of COVID-19 on Ecuador’s childhood vaccination program is crucial for developing targeted interventions to restore coverage levels and prevent future disruptions [18]. This analysis provides essential data for policymakers and public health officials working to strengthen immunization systems and improve pandemic preparedness [19].

The primary objective of this study is to quantify the impact of the COVID-19 pandemic on routine childhood vaccination coverage in Ecuador by comparing coverage rates before (ie, 2019) and during (ie, 2020‐2021) the pandemic and to identify geographical disparities in vaccination access during this period.

## Methods

### Study Design

This study used a retrospective, observational design to analyze vaccination coverage data from Ecuador’s national immunization program. We conducted a comparative analysis examining routine childhood vaccination coverage for the prepandemic period (2019) and the pandemic period (2020‐2021) [20]. This design allowed for the examination of temporal trends and changes in vaccination coverage, providing insights into the pandemic’s impact on immunization services.

### Data Sources and Collection

Primary data for this study were obtained from three key sources:

- Ministry of Public Health National Immunization Strategy Bulletin: This official source provided comprehensive vaccination coverage data at the national and provincial level, including the number of doses administered, target populations, and calculated coverage rates for all routine childhood vaccines [21].
- National Institute of Statistics and Censuses (INEC): INEC provided demographic data including population estimates, birth rates, and population projections used to calculate coverage rates and understand target populations [22].
- Published literature: To provide additional context and support for the findings, we conducted a systematic review of relevant peer-reviewed studies using PubMed, Scopus, and Web of Science databases with keywords including “COVID-19,” “vaccination coverage,” “Ecuador,” “childhood immunization,” and “pandemic impact” [23].

### Study Population

The study population consisted of children under 24 months of age in Ecuador, representing the target age group for routine childhood vaccinations according to the national immunization schedule [24]. Data were analyzed for all 24 provinces across 4 geographical regions: Costa (coast), Sierra (highlands), Amazonía (Amazon region), and Insular (Galápagos Islands) [25].

### Vaccination Coverage Metrics

We analyzed coverage for the following vaccines according to Ecuador’s national immunization schedule [26]:

- BCG: administered at birth
- Hepatitis B: first dose at birth
- Pentavalent (DTP-hepatitis B-*Haemophilus influenzae* type b): three doses at 2, 4, and 6 months
- Pneumococcal conjugate: three doses at 2, 4, and 6 months
- Inactivated poliovirus vaccine: two doses at 2 and 4 months
- Bivalent oral polio vaccine: doses at 6 and ≥12 months
- Rotavirus: two doses at 2 and 4 months
- Measles-mumps-rubella (MMR): two doses at 12 and 18 months
- Yellow fever: single dose at 12 months
- Varicella: single dose at 15 months
- DTP booster: fourth dose at 12‐15 months

Coverage rates were calculated as the percentage of children in the target age group who received the recommended number of doses for each vaccine, following World Health Organization (WHO) guidelines for vaccination coverage assessment [27].

### Data Analysis

Statistical analyses were performed using SPSS (version 28.0; IBM Corp) [28]. The following analytical approaches were used:

- Descriptive statistics: We calculated frequencies, percentages, means, and standard deviations to summarize vaccination coverage data and population characteristics [29].
- Comparative analysis: Coverage rates were compared between the prepandemic year (2019) and pandemic years (2020‐2021) using appropriate statistical methods. We calculated absolute and relative changes in coverage between time periods [30].
- Geographical analysis: We examined regional and provincial variations in vaccination coverage to identify areas most affected by pandemic-related disruptions [31].
- Trend visualization: Coverage data were plotted over time to visualize trends and identify patterns of decline or recovery across different vaccines and regions using the *matplotlib* and *seaborn* libraries in Python [32].

### Ethical Considerations

This study used secondary, publicly available data from official government sources and did not involve direct human subjects research. Therefore, ethical approval from an institutional review board was not required [33]. All data were anonymized and analyzed in aggregate form, ensuring privacy protection [34].

### Data Quality and Limitations

Data quality was ensured through cross-referencing between the Ministry of Public Health and INEC sources. Limitations include the lack of detailed socioeconomic data at the individual level and the absence of data beyond 2021, which would allow assessment of recovery efforts [35].

## Results

### Overall Vaccination Coverage Trends

Analysis of routine childhood vaccination data revealed a clear pattern of declining coverage between 2019 and 2021, demonstrating the significant impact of the COVID-19 pandemic on adherence to immunization schedules [36]. Table 1 presents comprehensive coverage data showing this concerning trend across all major vaccines.

**Table 1.** Regional and provincial population data for the years 2019, 2020, and 2021.^a^

Region and province		2019	2020	2021
Costa				
	Esmeraldas	13,293	13,211	13,128
	Manabí	29,299	29,207	29,005
	Los Ríos	18,897	18,888	18,798
	Santa Elena	8834	8897	8900
	Guayas	79,543	79,535	79,519
	Santo Domingo	10,535	10,537	10,541
	El Oro	12,526	12,464	12,438
Sierra				
	Azuay	15,903	15,700	15,688
	Bolívar	4338	4223	4205
	Cañar	5680	5660	5640
	Carchi	3258	3236	3214
	Cotopaxi	10,355	10,304	10,293
	Chimborazo	9853	9764	9660
	Imbabura	9173	9141	9115
	Loja	9978	9923	9872
	Pichincha	56,698	57,062	57,200
	Tungurahua	10,166	10,111	10,069
Amazonía				
	Morona Santiago	4895	4842	4822
	Napo	3341	3361	3381
	Orellana	3883	3821	3800
	Pastaza	2639	2659	2679
	Sucumbíos	4944	4958	4978
	Zamora Chinchipe	2839	2837	2833
Insular				
	Galápagos	624	631	666
Total		331,494	330,972	330,444

a Dirección Nacional de Estadística y Análisis de Información de Salud (DNEAIS), early and late capture base, developed by the Ministry of Public Health National Immunization Strategy.

**Figure 1.**
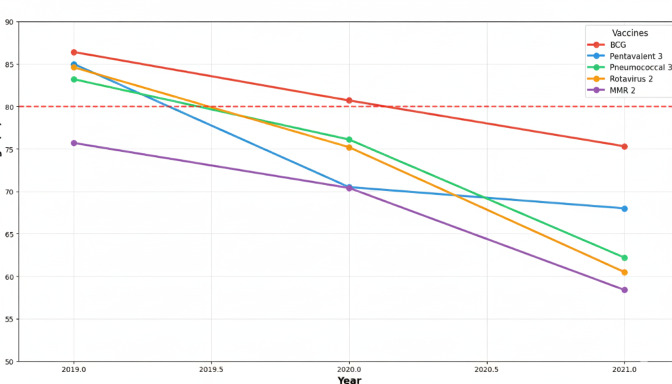
Vaccination coverage trends in Ecuador (2019-2021). The line graph shows the temporal trends for BCG, pentavalent 3, pneumococcal 3, rotavirus 2, and MMR 2 vaccines, with the 80% World Health Organization threshold line. MMR: measles-mumps-rubella.

**Figure 2.**
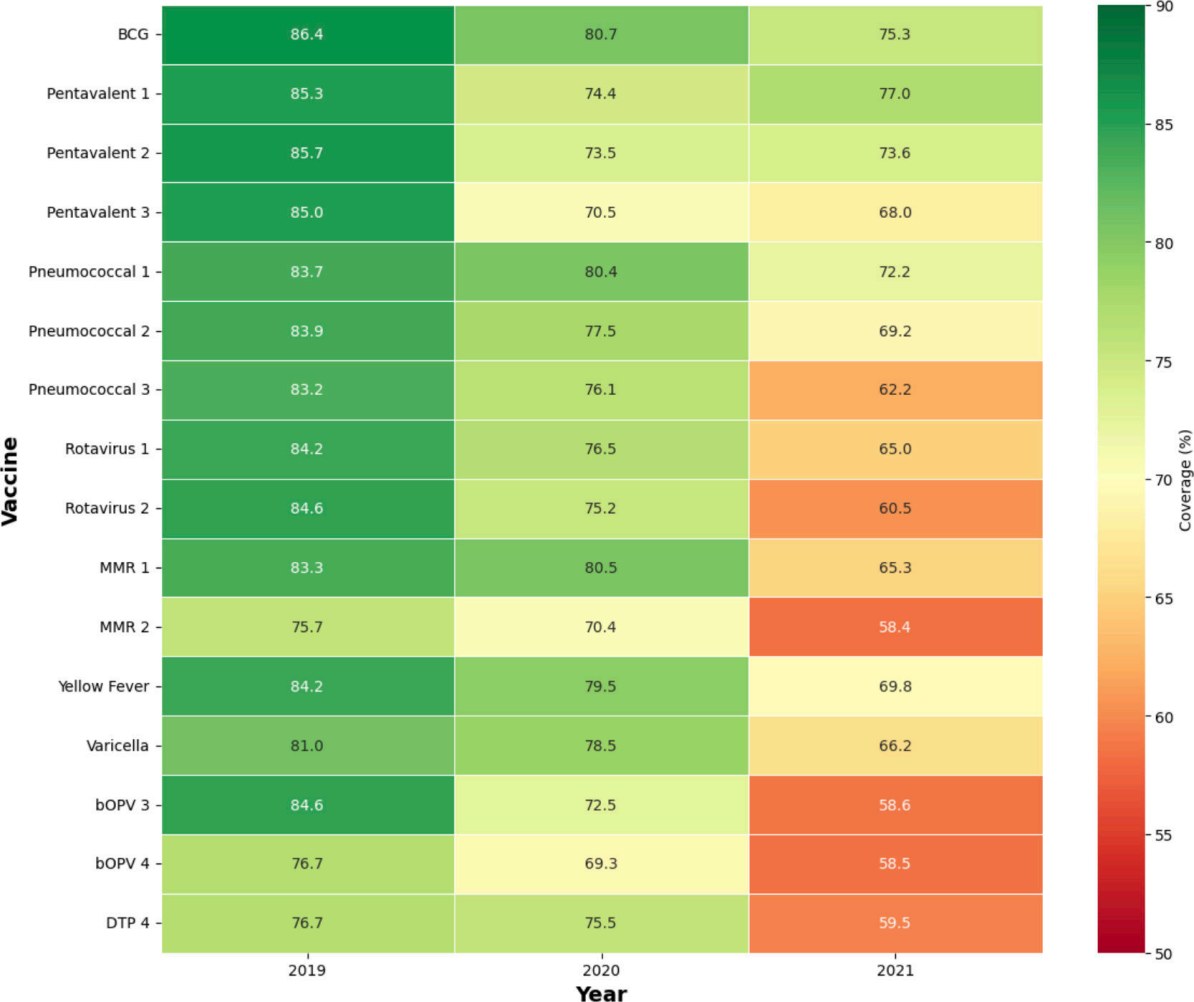
Heatmap of vaccination coverage by vaccine and year. A color-coded heatmap showing all vaccines across the 3 years, with coverage percentages displayed. bOPV: bivalent oral polio vaccine; DTP: diphtheria-pertussis-tetanus; MMR: measles-mumps-rubella.

Figure 1 illustrates the temporal trends in vaccination coverage for key vaccines from 2019 to 2021. The visualization clearly demonstrates the progressive decline in coverage rates, with the most dramatic decreases occurring between 2020 and 2021. A heatmap provides an alternative visualization of the comprehensive data presented in Table 2, highlighting the widespread nature of the coverage decline (Figure 2).

**Table 2.** Vaccination coverage by target group, vaccine type, and year (2019-2021).^a^

Target group and vaccine		2019		2020		2021	
		Doses applied	Coverage, %	Doses applied	Coverage, %	Doses applied	Coverage, %
Birth (4 h)							
	BCG total	286,569	86.4	266,961	80.7	248,812	75.3
	HB^b^ zero	237,145	71.5	204,979	61.9	202,679	61.3
2 months							
	Pentavalent 1	282,623	85.3	246,141	74.4	254,565	77
	Pneumococcal 1	277,310	83.7	265,924	80.4	238,605	72.2
	IPV^c^ 1	282,277	85.2	263,867	79.7	232,631	70.4
	Rotavirus 1	278,994	84.2	253,192	76.5	214,668	65
4 months							
	Pentavalent 2	284,078	85.7	243,317	73.5	243,082	73.6
	Pneumococcal 2	278,085	83.9	256,408	77.5	228,686	69.2
	IPV 2	282,171	85.1	260,538	78.7	211,797	64.1
	Rotavirus 2	280,431	84.6	248,973	75.2	199,909	60.5
6 months							
	Pentavalent 3	281,734	85	233,371	70.5	224,702	68
	Pneumococcal 3	275,947	83.2	251,977	76.1	205,659	62.2
	bOPV^d^ 3	280,390	84.6	239,889	72.5	193,510	58.6
12 months							
	MMR 1	276,289	83.3	266,550	80.5	215,874	65.3
	Yellow fever	279,008	84.2	263,123	79.5	230,524	69.8
15 months							
	Varicella	268,434	81	259,880	78.5	218,800	66.2
18 months							
	MMR^e^ 2	250,964	75.7	232,883	70.4	192,835	58.4
1 year from third dose							
	bOPV 4	254,395	76.7	229,210	69.3	193,234	58.5
	DTP^f^ 4	254,256	76.7	249,857	75.5	196,616	59.5

a Dirección Nacional de Estadística y Análisis de Información de Salud (DNEAIS), early and late capture base, developed by the Ministry of Public Health national immunization strategy.

b HB: hepatitis B.

c IPV: inactivated poliovirus vaccine.

d bOPV: bivalent oral polio vaccine.

e MMR: measles-mumps-rubella.

f DTP: diphtheria-pertussis-tetanus.

### Vaccine-Specific Coverage Analysis

**Figure 3.**
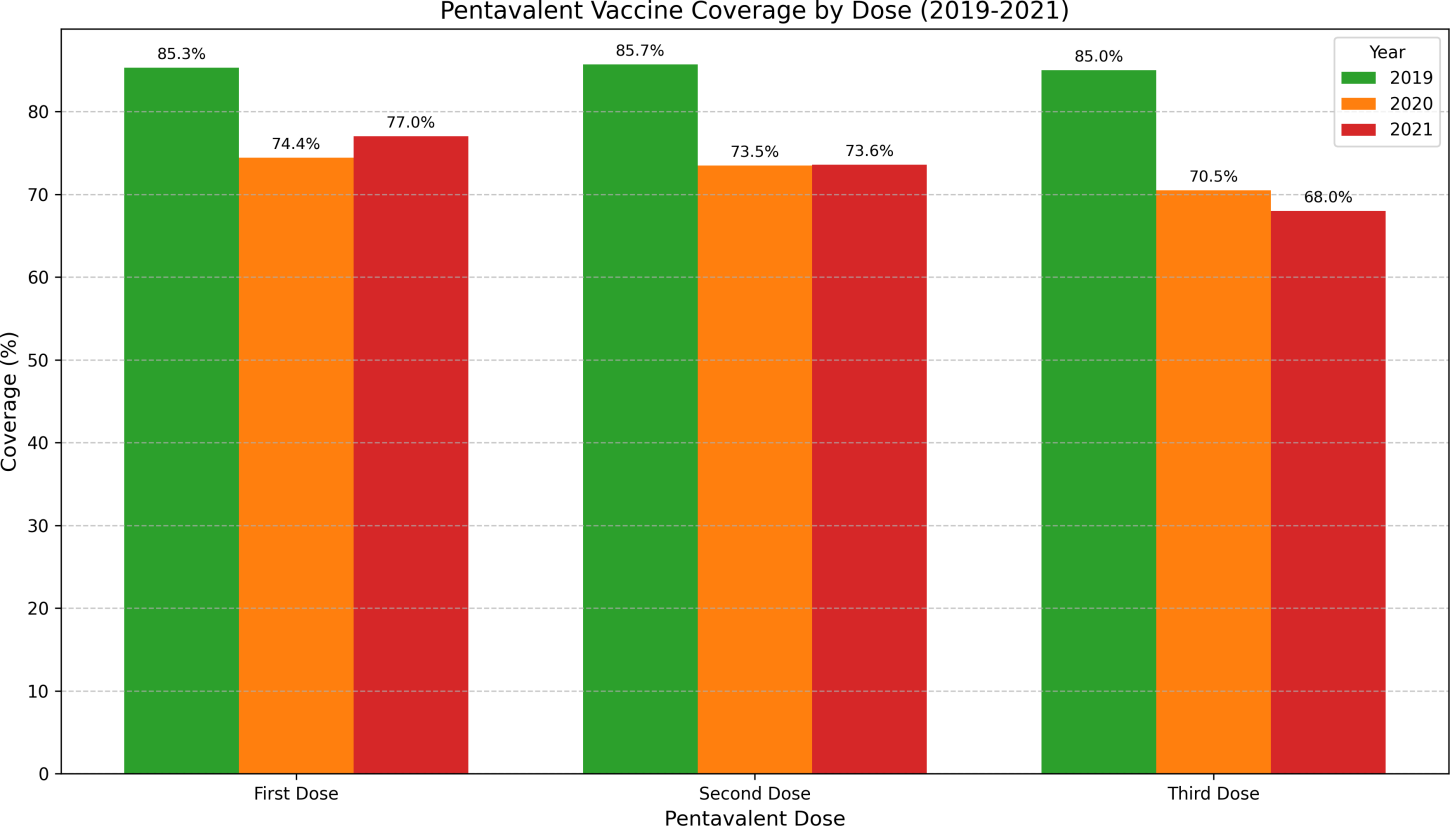
Pentavalent series coverage by dose and year. Bar chart comparing coverage rates for the 3 pentavalent doses across years.

We conducted an analysis on vaccine-specific coverage and found the following:

- BCG vaccine (birth): Coverage for BCG, administered at birth, decreased progressively from 86.4% in 2019 (286,569 doses) to 80.7% in 2020 (266,961 doses) and further to 75.3% in 2021 (248,812 doses). This represents a cumulative decrease of 11.1 percentage points over the 2-year period [37].
- Pentavalent vaccine series: The pentavalent vaccine showed variable patterns across doses. First dose coverage declined from 85.3% in 2019 to 74.4% in 2020 but showed slight recovery to 77% in 2021. However, completion rates for the 3-dose series remained substantially below prepandemic levels, with third dose coverage falling from 85% in 2019 to 68% in 2021 [38]. This growing gap between initiation and completion of the series is a critical indicator of service disruption (Figure 3).
- Pneumococcal vaccine: This vaccine experienced consistent declines across all 3 doses. First dose coverage fell from 83.7% in 2019 to 72.2% in 2021, while third dose coverage dropped more dramatically from 83.2% to 62.2% over the same period [39].
- Rotavirus vaccine: Among the most affected vaccines, rotavirus coverage showed severe declines. Second dose coverage plummeted from 84.6% in 2019 to 60.5% in 2021, representing a 24.1 percentage point decrease [40].
- MMR: MMR vaccine coverage demonstrated significant drops, particularly for the second dose administered at 18 months. Coverage fell from 75.7% in 2019 to 58.4% in 2021, indicating potential vulnerability to measles outbreaks [41].

### Regional and Provincial Disparities

**Figure 4.**
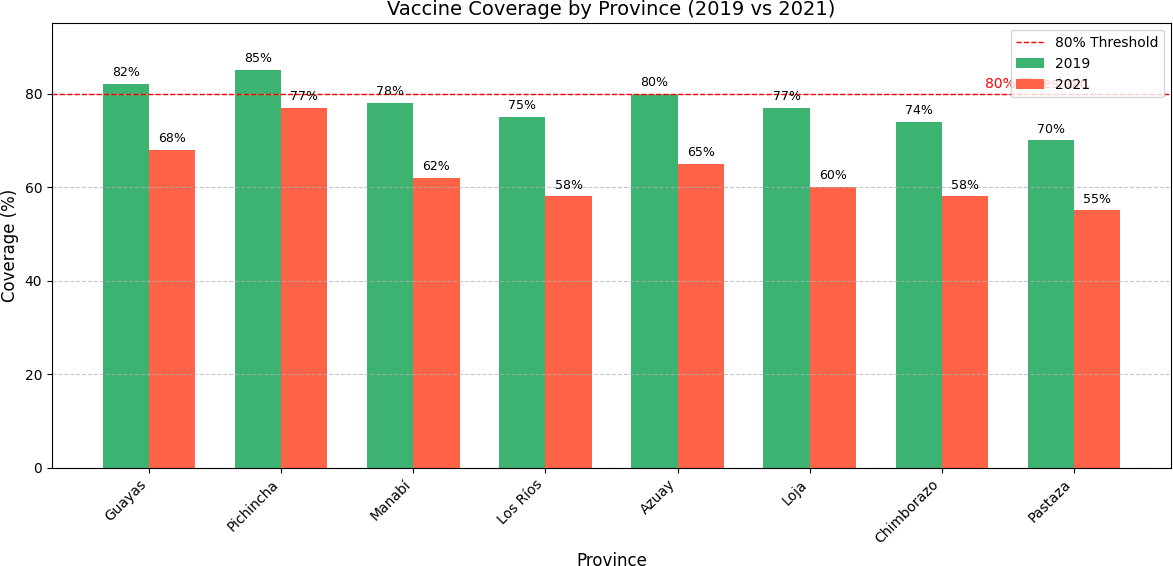
Regional comparison of vaccine coverage in 2019 and 2021.

Table 2 presents population data across Ecuador’s 4 main regions and 24 provinces, providing context for understanding vaccination disparities. Analysis revealed significant geographical variations in pandemic impact [42] (Figure 4):

- Coastal region (Costa): This region, including major urban centers like Guayas (containing Guayaquil), experienced substantial coverage declines. Rural coastal provinces such as Esmeraldas and Los Ríos showed particularly severe disruptions [43].
- Highland region (Sierra): Provincial coverage varied significantly, with Pichincha (containing Quito) maintaining relatively better coverage compared to rural provinces like Bolívar and Cañar [44].
- Amazon region (Amazonía): These provinces, already facing geographical access challenges, experienced compounded difficulties during the pandemic. Remote provinces like Pastaza and Zamora Chinchipe showed marked coverage declines [45].
- Galápagos (Insular): Despite its small population, this region maintained relatively stable coverage due to its isolated nature and focused health interventions [46].

### Magnitude of Coverage Loss

Comparative analysis revealed that approximately 137,000 fewer vaccine doses were administered in 2020 compared to 2019, with further decreases in 2021 [47]. The pentavalent vaccine showed the most substantial absolute reduction (17.7%), followed by poliovirus, rotavirus, and pneumococcal vaccines [48].

### Public Health Implications

The sustained decline in vaccination coverage through 2021 indicates that pandemic effects on childhood immunization were not temporary disruptions but represented persistent challenges to the health system [49]. Coverage rates falling below critical thresholds (particularly those below 80%) increase the risk of vaccine-preventable disease outbreaks, especially in areas with clustered susceptible populations [50].

## Discussion

### Principal Findings and Global Context

Our findings reveal a concerning pattern of declining routine childhood vaccination coverage in Ecuador during the COVID-19 pandemic, with sustained decreases through 2021. These results align with global trends documented worldwide, where the pandemic disrupted essential health services beyond the direct impact of SARS-CoV-2 infection [51]. The magnitude of decline in Ecuador—with some vaccines showing coverage drops exceeding 20 percentage points—represents one of the more severe impacts documented in Latin America [52].

The observed patterns are consistent with findings from other Latin American countries. Castro-Aguirre et al’s [9] regional analysis of 39 countries showed significant reductions in DTP vaccine coverage in 79% of assessed regions, with Ecuador among the most affected. Our data add valuable country-specific detail to this regional picture, demonstrating the heterogeneous impact across different vaccines and geographical areas [53].

### Factors Contributing to Coverage Decline

Several interconnected factors contributed to the vaccination coverage declines observed in Ecuador [54]:

- Health care system disruptions: The reallocation of health care resources to COVID-19 response efforts, including health care worker deployment to the pandemic response, reduced capacity for routine services [55]. Many health facilities were repurposed for COVID-19 care or experienced reduced operating capacity due to infection control measures [56].
- Movement restrictions and access barriers: Government-imposed lockdowns and movement restrictions, particularly strict during Ecuador’s initial pandemic response, limited families’ ability to access vaccination services [57]. Rural populations faced compounded challenges with transportation disruptions [58].
- Fear of infection: Parents’ concerns about COVID-19 exposure in health care settings led to delayed or avoided vaccination appointments [59]. This behavioral factor persisted even as restrictions were lifted, contributing to continued coverage declines in 2021 [60].
- Supply chain disruptions: Global and regional supply chain disruptions affected vaccine availability and distribution, though specific vaccine stockout data were not consistently available for this analysis [61].

### Regional Disparities and Equity Concerns

The geographical analysis revealed significant disparities in pandemic impact across Ecuador’s regions [62]. Coastal and highland provinces experienced the most severe reductions, while some Amazon provinces showed variable patterns. These disparities reflect preexisting inequalities in health care access that were exacerbated during the pandemic [63].

Urban centers like Quito and Guayaquil, despite having better health care infrastructure, experienced substantial coverage declines, likely due to higher COVID-19 transmission concerns and stricter lockdown measures [64]. Rural provinces faced the dual challenge of limited health care access and additional pandemic-related barriers [65].

Indigenous and rural populations, who already faced coverage gaps before the pandemic, were disproportionately affected [66]. Arce Becerra et al’s [42] study of Quito’s metropolitan district showed stark urban-rural differences, with rural parish coverage declining more severely than that in urban areas.

### Implications for Child Health and Disease Outbreaks

The sustained decline in vaccination coverage has serious implications for child health in Ecuador [67]. Coverage levels below 80% for most vaccines place the population at risk of vaccine-preventable disease outbreaks [68]. Of particular concern are the following:

- Measles risk: With MMR second dose coverage falling to 58.4% in 2021, Ecuador faces increased susceptibility to measles outbreaks, especially given the highly contagious nature of the measles virus and the WHO recommendation of 95% coverage for herd immunity [69].
- Pertussis and diphtheria: Declining pentavalent coverage increases the risk of these serious bacterial infections, which are particularly dangerous in young infants who rely on maternal antibodies and community immunity [70].
- Poliovirus: Although Ecuador has maintained polio-free status since 1990, reduced oral polio vaccine coverage raises concerns about potential importation and circulation of poliovirus, particularly given regional polio cases in neighboring countries [71].

### Recovery Strategies and Policy Recommendations

Addressing the vaccination coverage decline requires comprehensive, multifaceted interventions [72]:

- Catch-up vaccination campaigns: Targeted mass vaccination campaigns should prioritize children who missed routine vaccinations during the pandemic. Age-appropriate catch-up schedules need implementation to ensure complete immunization, following WHO catch-up vaccination guidelines [73].
- Health system strengthening: Investment in robust health systems that can maintain essential services during emergencies is crucial. This includes adequate staffing, infrastructure improvements, and emergency preparedness protocols [74].
- Community engagement and education: Addressing vaccine hesitancy through community-based education programs, particularly targeting misinformation about COVID-19 and routine vaccines, is essential for coverage recovery [75].
- Digital health innovations: Implementation of digital vaccination registries and reminder systems can improve tracking and follow-up of children requiring catch-up vaccinations [76].
- Integrated service delivery: Combining routine vaccination with other child health services and COVID-19 vaccination efforts can improve efficiency and access [77].

### Pandemic Preparedness and Resilience

Lessons learned from Ecuador’s experience should inform pandemic preparedness for future health emergencies [78]:

- Essential service designation: Routine childhood vaccination should be explicitly designated as essential during health emergencies, with specific protocols to maintain service delivery [79].
- Flexible service delivery models: Developing outreach vaccination programs and mobile clinics can ensure continued access during movement restrictions [80].
- Community health workers: Training and deploying community health workers for vaccination education and basic immunization services can maintain coverage in remote areas [81].

### Comparison With Global Recovery Efforts

International experience suggests that recovery of vaccination coverage requires sustained effort and multiple strategies. Countries like Rwanda and Bangladesh have demonstrated successful catch-up campaigns using innovative approaches including door-to-door vaccination and integration with COVID-19 vaccine delivery [82,83].

### Study Limitations

Several limitations should be acknowledged in interpreting these findings [84]:

- Temporal scope: The analysis is limited to 2019‐2021, preventing assessment of recovery efforts that may have begun in 2022‐2023 [85].
- Socioeconomic data: Detailed individual-level socioeconomic data were not available, limiting the ability to fully analyze equity impacts [86].
- Causal attribution: While temporal associations are clear, directly attributing all coverage changes to COVID-19 requires careful consideration of other concurrent factors [87].
- Subnational granularity: Provincial-level analysis, while informative, may mask important local variations within provinces [88].
- Administrative versus survey data: This study relies on administrative data, which may differ from population-based survey estimates of vaccination coverage [89].

### Future Research Directions

Future research should examine [90]:

- Recovery patterns in vaccination coverage post-2021
- Cost-effectiveness of different catch-up vaccination strategies
- Long-term impacts on vaccine-preventable disease incidence
- Specific interventions implemented to restore coverage
- Socioeconomic determinants of vaccination coverage disparities

### Conclusions

The COVID-19 pandemic profoundly impacted routine childhood vaccination coverage in Ecuador, with declines persisting through 2021. The evidence demonstrates that, while the immediate focus on the pandemic response was necessary, the collateral damage to essential health services created new public health challenges requiring urgent attention [91].

The sustained decline in vaccination coverage—with some vaccines showing decreases exceeding 20 percentage points—places Ecuador’s children at increased risk of vaccine-preventable disease outbreaks [92]. Regional disparities highlight how the pandemic exacerbated existing health inequities, with vulnerable populations facing compounded challenges in accessing immunization services [93].

Recovery requires comprehensive strategies addressing both immediate catch-up vaccination needs and longer-term health system strengthening [94]. Priority actions include implementing targeted mass vaccination campaigns, strengthening routine immunization services, and developing more resilient health systems capable of maintaining essential services during future health emergencies [95].

The findings underscore the critical importance of maintaining routine immunization programs during health crises and the need for pandemic preparedness plans that explicitly protect essential health services [96]. As Ecuador works to rebuild and strengthen its immunization program, the lessons learned from this pandemic experience must inform strategies to ensure no child is left unprotected against vaccine-preventable diseases [97].

Continued monitoring, evaluation, and research are essential to track recovery progress, evaluate intervention effectiveness, and inform evidence-based strategies for achieving and maintaining optimal vaccination coverage [98]. The protection of children’s health through sustained immunization programs remains a cornerstone of public health that must be safeguarded against future disruptions [99].

## Abbreviations

DTP: diphtheria-pertussis-tetanus
INEC: National Institute of Statistics and Censuses
MMR: measles, mumps, and rubella
WHO: World Health Organization
